# The “Typical” Asian Diet Is Anything But: Differences in Dietary Exposure to Metals among Subgroups of U.S. Asians

**DOI:** 10.1289/ehp.125-A58

**Published:** 2017-03-01

**Authors:** Lindsey Konkel

**Affiliations:** Lindsey Konkel is a New Jersey–based journalist who reports on science, health, and the environment.

The U.S. Asian population consists of many culturally and geographically diverse ethnic subgroups. In nationally representative population-based samples, these groups often are lumped together in race categories such as “Asian” or “Other,” which can mask differences in the ways that environmental exposures affect health in subpopulations. In a pair of new studies in *EHP*, researchers assess diet, dietary intake of metals, and associated biomarker concentrations across three Asian subgroups.^1^ ^,^ ^2^

**Figure:**
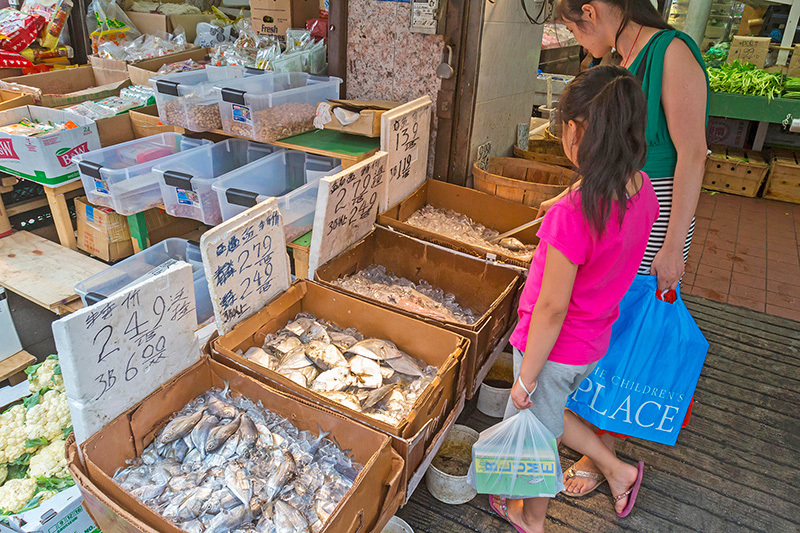
Although various U.S. Asian subgroups share some similarities in the foods they typically eat, there are also enough dissimilarities to make a difference in diet-related exposures. © Richard Green/Alamy Stock Photo

A 2014 analysis by the Centers for Disease Control and Prevention (CDC) found that U.S. Asians overall tended to have higher levels of several metals and metalloids in their blood and urine than white, black, or Hispanic populations.^3^ These substances have been linked to cancer, heart disease, developmental defects, damage to the nervous system, and kidney failure.^4^ ^,^ ^5^ ^,^ ^6^ ^,^ ^7^ However, the 2014 analysis was unable to assess whether exposures varied among Asian subgroups. Such distinctions could have important implications for preventing and treating disease.^8^

Asian Americans make up about 6% of the U.S. population.^9^ If researchers sampled Asian Americans in proportion to their total population, just 6 out of every 100 research participants would be Asian. That may not be a large enough sample to draw out statistically significant differences among ethnic Asian subgroups.

In the 2011–2012 iteration of its National Health and Nutrition Examination Survey (NHANES), the CDC oversampled Asians, meaning the surveyors included a greater number of Asians in proportion to their representation in the U.S. population. They also collected demographic information on three Asian subgroups: Chinese and Asian Indian—each accounting for about 20% of the U.S. Asian population—as well as “Other Asian,” a group that census figures indicate is composed mainly of Filipino, Vietnamese, Korean, and Japanese Americans.^1^

“It’s a big deal that NHANES did this, because it gives us an unprecedented opportunity to study in depth the national health profile of a particular group of people,” says Moon Chen, an expert in cancer health disparities who works at the University of California, Davis. Chen was not involved with the new research.

In one of the two studies, researchers at The University of Texas Health Science Center at Houston (UTHealth) used NHANES data to compare blood and urine levels of lead, mercury, arsenic, and cadmium among the three Asian subgroups. They found that levels of mercury, arsenic, and cadmium were similar among Chinese and Other Asians, but lower among Asian Indians. Asian Indians, however, had slightly higher blood lead levels than the other two groups.^1^

The researchers posit that these higher blood lead levels could have been associated with Asian Indians’ common use of certain spices and cosmetics, since elevated levels of lead have been found in the spice turmeric^10^ ^,^ ^11^ and in eye makeup known as kohl, or surma.^12^ U.S.-born Asians generally had lower average levels of all four metals than Asians born outside the United States.^1^

Sex, age, education level, smoking status, and fish consumption also predicted biomarker levels of specific metals in some Asian subgroups. Food consumption is known to be a major source of exposure to mercury, arsenic, and cadmium in the general population. Among the Asian populations, fish contributed the most mercury and total arsenic, while rice contributed the most inorganic arsenic, which is the more toxic form of the metalloid. Vegetables, cereal grains, fruits, and dairy products contributed the most cadmium among nonsmokers (tobacco is a major source of this metal).^1^

In the second study, the researchers compared the three Asian subgroups with other U.S. racial/ethnic groups in terms of food consumption and associated metal intake levels.^2^ They combined data from the NHANES dietary questionnaire, the U.S. Environmental Protection Agency’s Food Commodity Intake Database, and the U.S. Food and Drug Administration’s Total Dietary Study to estimate people’s intake of metals via food. The team found that blood and urine levels of mercury and both total and inorganic arsenic were significantly associated with estimated dietary metal intake among Asians. There were no associations between dietary intake and blood levels of either lead or cadmium.

As a whole, Asians had the highest fish and rice consumption among U.S. racial/ethnic groups, eating on average more than twice as much fish and rice daily as whites. Asian Indians ate less fish than Chinese and Other Asians, while levels of rice consumption were similar among the subgroups.^2^

“Our study results confirm that fish and rice are important dietary sources of mercury and arsenic exposure for these groups,” says lead author Hiroshi Awata, an environmental toxicologist who conducted the research while at UTHealth School of Public Health. “As the U.S. population becomes more diverse, it’s important to understand specific dietary patterns for smaller ethnic groups and how that might relate to health status.”

“The two papers leverage a wealth of data from NHANES, USDA, and FDA to provide very useful estimates of the dietary intake of toxic metals, and link them to biomarker levels of metals among U.S. Asians,” says Youssef Oulhote, a research fellow in environmental health at the Harvard T.H. Chan School of Public Health. “This points to a cumulative burden of toxic metals exposures among this specific population.” Oulhote, who was not involved in the new studies, previously published work suggesting that Asian populations also may have higher manganese levels.^13^

The Total Dietary Study did not provide data on metal concentrations for some of the foods that are regularly eaten by certain Asian subgroups, such as seaweed. And it is not clear what role geography—specifically, where a particular food was caught or farmed—may have had in determining exposure levels. Awata says future studies are needed to address these questions and probe associations between socioeconomic status and biomarker levels—for instance whether subsistence fishing leads to higher levels of mercury contamination in economically disadvantaged Asian subgroups.
